# Compartmentalized cAMP Signaling Associated With Lipid Raft and Non-raft Membrane Domains in Adult Ventricular Myocytes

**DOI:** 10.3389/fphar.2018.00332

**Published:** 2018-04-23

**Authors:** Shailesh R. Agarwal, Jackson Gratwohl, Mia Cozad, Pei-Chi Yang, Colleen E. Clancy, Robert D. Harvey

**Affiliations:** ^1^Department of Pharmacology, University of Nevada, Reno, Reno, NV, United States; ^2^Department of Pharmacology, University of California, Davis, Davis, CA, United States

**Keywords:** cAMP, compartmentation, membrane microdomains, lipid rafts, caveolae

## Abstract

**Aim:** Confining cAMP production to discrete subcellular locations makes it possible for this ubiquitous second messenger to elicit unique functional responses. Yet, factors that determine how and where the production of this diffusible signaling molecule occurs are incompletely understood. The fluid mosaic model originally proposed that signal transduction occurs through random interactions between proteins diffusing freely throughout the plasma membrane. However, it is now known that the movement of membrane proteins is restricted, suggesting that the plasma membrane is segregated into distinct microdomains where different signaling proteins can be concentrated. In this study, we examined what role lipid raft and non-raft membrane domains play in compartmentation of cAMP signaling in adult ventricular myocytes.

**Methods and Results:** The freely diffusible fluorescence resonance energy transfer-based biosensor Epac2-camps was used to measure global cytosolic cAMP responses, while versions of the probe targeted to lipid raft (Epac2-MyrPalm) and non-raft (Epac2-CAAX) domains were used to monitor local cAMP production near the plasma membrane. We found that β-adrenergic receptors, which are expressed in lipid raft and non-raft domains, produce cAMP responses near the plasma membrane that are distinctly different from those produced by E-type prostaglandin receptors, which are expressed exclusively in non-raft domains. We also found that there are differences in basal cAMP levels associated with lipid raft and non-raft domains, and that this can be explained by differences in basal adenylyl cyclase activity associated with each of these membrane environments. In addition, we found evidence that phosphodiesterases 2, 3, and 4 work together in regulating cAMP activity associated with both lipid raft and non-raft domains, while phosphodiesterase 3 plays a more prominent role in the bulk cytoplasmic compartment.

**Conclusion:** These results suggest that different membrane domains contribute to the formation of distinct pools of cAMP under basal conditions as well as following receptor stimulation in adult ventricular myocytes.

## Introduction

The diffusible second messenger cAMP mediates responses to a wide array of neurotransmitters, hormones, and autacoids acting through a variety of GPCRs in virtually every cell in the human body. A partial list of cellular functions regulated by cAMP include gene expression, glucose and lipid metabolism, steroidogenesis, insulin secretion, fluid and electrolyte secretion, muscle contraction, muscle relaxation, as well as nerve and muscle electrical excitability (Robison et al., 1968; Gancedo, 2013). What is even more remarkable is that even though cAMP regulates multiple processes in any given cell, receptor dependent stimulation of cAMP production often elicits unique downstream responses. The classic example is in cardiac myocytes, where both βARs and EPRs stimulate cAMP production. Yet only the cAMP produced by βARs regulates the electrical and mechanical properties of these cells (Buxton and Brunton, 1983; Steinberg and Brunton, 2001). Observations such as this led to the idea that cAMP signaling must be compartmentalized.

One important factor in maintaining the fidelity of receptor-mediated responses is the formation of signaling complexes that organize effectors of cAMP, such as PKA, together with the target proteins they regulate. This often occurs through interactions with scaffolding proteins like AKAPs. Numerous studies have demonstrated that disrupting AKAP interactions can alter cAMP responses (Scott et al., 2013). However, signaling complexes alone are not sufficient to explain compartmentation. If stimulation of every receptor produced a uniform increase in cAMP throughout the cell, they would all elicit the same responses. Therefore, there must also be mechanisms for creating discrete, localized pools of cAMP.

Much progress has been made in identifying how cAMP compartmentation occurs, due in no small part to the development of genetically encoded biosensors that can be used to study responses in different subcellular locations of live cells (Nikolaev and Lohse, 2006; Berrera et al., 2008). Using these tools, many studies have focused on the role of PDEs, which breakdown cAMP and are commonly thought to act as functional barriers that define different signaling domains (Brunton, 2003; Fischmeister et al., 2006; Conti et al., 2014). However, modeling studies have argued that while PDE activity is necessary, it alone is not sufficient (Rich et al., 2000, 2001; Saucerman et al., 2006, 2014; Iancu et al., 2007, 2008; Feinstein et al., 2012; Agarwal et al., 2014). In addition to the non-uniform distribution of PDE activity, unique receptor-dependent responses can only be explained if cAMP is produced in distinctly different physical locations.

The fluid mosaic model originally proposed that proteins diffuse freely in the plasma membrane (Singer and Nicolson, 1972) and that signal transduction occurs through random interactions of these proteins (Tolkovsky and Levitzki, 1978). However, it is now known that the density of signaling proteins is too low for this to be effective. Furthermore, the movement of membrane proteins has been shown to be restricted by various means (Bethani et al., 2010), suggesting that the plasma membrane is segregated into distinct microdomains where different signaling proteins can be concentrated. One such mechanism for achieving this is through the formation of liquid-order domains called lipid rafts, which are characterized by the presence of cholesterol and sphingolipids (Jacobson et al., 2007). Membrane fractionation studies have demonstrated that certain receptor proteins are concentrated in these detergent resistant membrane fractions, while others are specifically excluded (Brown, 2006; Allen et al., 2007).

Both biochemical and functional studies support the notion that caveolae, a specific subset of lipid rafts associated with the scaffolding protein caveolin, play a particularly important role in generating compartmentalized cAMP responses in cardiac myocytes (Rybin et al., 2000; Head et al., 2005; Harvey and Calaghan, 2012). Consistent with this idea, it has been previously demonstrated that disrupting lipid rafts by cholesterol depletion selectively alters cAMP responses associated with receptors believed to reside in caveolar lipid rafts (Rybin et al., 2000; Head et al., 2005; Agarwal et al., 2011). Using FRET-based biosensors targeted to lipid raft and non-raft domains of the plasma membrane we demonstrate that it is possible to detect differences in cAMP signaling associated with these specific membrane environments in adult cardiac myocytes. The results have important implications with respect to how cAMP responses are coordinated. They also make the often-overlooked point that the plasma membrane is not homogenous and when using membrane-targeted probes it is important to consider their exact location.

## Materials and Methods

### Cell Isolation and Culture

Ventricular myocytes were isolated from the hearts of male Sprague Dawley rats (250–300 g) using a modification of the procedure previously described (Agarwal et al., 2011). Rats were anesthetized with a pentobarbital injection (150 mg/kg i.p.). The hearts were quickly excised and myocytes isolated. The protocol used was in accordance with the *Guide for the Care and Use of Laboratory Animals* as adopted by National Institutes of Health and approved by the Institutional Animal Care and Use Committee at the University of Nevada, Reno. Myocytes used for FRET and confocal imaging experiments were resuspended and plated in minimum essential medium (MEM) containing insulin-transferrin-selenium (1X), bovine serum albumin (1 mg/ml), 2,3-butanedione monoxime (10 mM) and penicillin-streptomycin. After incubation for 2 h, the cells were transduced with adenovirus constructs expressing different biosensors as described previously (Warrier et al., 2005, 2007; Agarwal et al., 2011). Imaging experiments were conducted 48–72 h after infection. We have previously reported that, while there is some loss of cholesterol content, cells maintained in culture under these conditions do not exhibit a marked loss in caveolae at cell surface, and compartmentalized cAMP responses associated with membrane microdomains are preserved (Agarwal et al., 2011; Macdougall et al., 2012). All experiments were carried out in extracellular solution containing (in mM): NaCl 137, KCl 5.4, MgCl_2_ 0.5, CaCl_2_ 1.0, NaH_2_PO_4_ 0.33, HEPES 5, glucose 5.5, pH 7.4, at room temperature.

**FIGURE 1:**
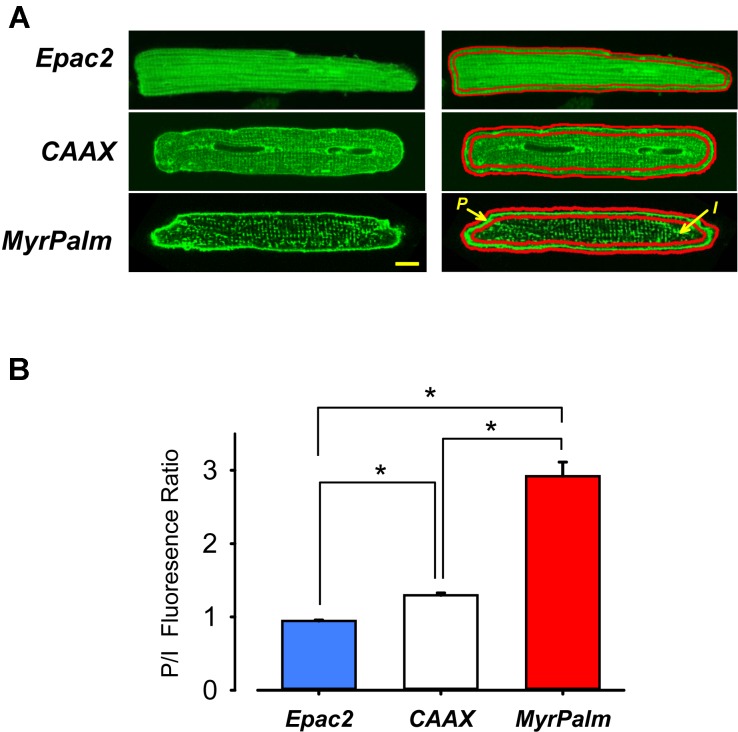
Targeting of Epac2-based FRET biosensors to different subcellular locations in adult ventricular myocytes. **(A)** Confocal images of adult rat ventricular myocytes expressing Epac2-camps (Epac2), Epac2-CAAX (CAAX), and Epac2-MyrPalm (MyrPalm). Average fluorescence intensity was measured at the periphery (P) and in the interior (I) of cells (as illustrated in the right hand panels) to obtain P/I fluorescence intensity ratio as shown. Scale bar: 10 μm. **(B)** Summary of average P/I ratio in cells expressing Epac2 (blue bar), CAAX (white bar), and MyrPalm (red bar). The significant difference (^∗^*p* < 0.001, n/N = 21/7, Epac2; 22/8, CAAX; 10/4, MyrPalm; Kruskal–Wallis one-way ANOVA on Ranks followed by Dunn’s test for pairwise multiple comparisons) in the P/I fluorescence intensity ratios for the different biosensors demonstrate that Epac2-MyrPalm expression is more prominent in the peripheral plasma membrane, while Epac2-CAAX is more uniformly expressed in the plasma membrane throughout the cell, including the t-tubules.

Experiments were conducted using Epac2-camps, a FRET-based biosensor that lacks any targeting sequences and is expressed uniformly throughout the cytosolic compartment of cells (Nikolaev et al., 2004; Iancu et al., 2008). We also used versions of this probe targeted to lipid raft (Epac2-MyrPalm) or non-raft (Epac2-CAAX) domains of the plasma membrane (Agarwal et al., 2014). For confocal imaging, myocytes expressing the different probes were washed and resuspended in extracellular solution before transferring to 35 mm glass-bottom fluorodishes (World Precision Instruments, Inc.). Confocal imaging was performed on an Olympus Fluoview 1000 microscope using an argon laser (515 nm line) to excite eYFP (Agarwal et al., 2014). Images were exported as tiff files, and the contrast and brightness of these images were adjusted in ImageJ software for presentation purposes. How much of each FRET-based probe was targeted to the peripheral plasma membrane as opposed to t-tubule membranes in the interior of the cell was determined by drawing two regions of interest (ROI), as shown in **Figure 1A**. We then calculated the ratio of the average background-subtracted fluorescence intensity of these two areas.

### Fluorescence Recovery After Photobleaching (FRAP)

Experiments were conducted using the protocol described previously (Agarwal et al., 2014). Briefly, a circular region 5 μm in diameter was drawn on the peripheral sarcolemma (for the membrane-targeted probes) or the middle of the cell, away from nucleus (for the cytosolic biosensor). This area was then bleached using the 515 nm line of the argon laser at full power. For the membrane-associated cAMP sensors, a 512 × 512 pixel window with 1.2X zoom and 2 μs pixel time were used to collect the images before and after the bleaching using a laser intensity of 1–2%. These setting allowed us to collect individual images every 1.5 s. FRAP experiments involving membrane targeted probes were also conducted by bleaching regions in the interior of the cell to verify that the results were the same. Due to faster movement of the freely-diffusible Epac2-camps sensor, images were collected from a region 128 × 128 pixels, allowing a frame rate of ∼0.18 s, which is fast enough to monitor the recovery of cytosolic proteins (Day et al., 2012). Collection of images at a faster frame rate of 0.09 s (64 × 64 pixels) gave similar fluorescence recovery values (data not shown). Fluorescence recovery curves were generated by plotting recovery of relative fluorescence in the bleached area as a function of time. The mobile fraction (M_f_) and fluorescence recovery half time (t_1/2_) were calculated as described (Reits and Neefjes, 2001) using SigmaPlot software. The M_f_ was calculated using the formula: *M_f_* = (*F_∞_ – F*_0_)/(*F*_i_ – *F*_0_) where *F_∞_* is the fluorescence in the bleached region upon full recovery, F_0_ is fluorescence just after photobleaching, and *F_i_* is the fluorescence before bleaching. The t_1/2_ was calculated as the time required for the fluorescence intensity to recover to 50% of *F_∞_*.

### Fluorescence Resonance Energy Transfer

Experiments were carried out using intact myocytes expressing the various Epac2-based biosensors, as described previously (Warrier et al., 2005, 2007; Agarwal et al., 2011). Images were recorded on the stage of an inverted microscope (Olympus IX71) using an OrcaD2 dual chip CCD camera and HCImage data acquisition and analysis software (Hamamatsu, Inc.). Changes in cAMP activity were defined as the change in background and bleed-through corrected eCFP/eYFP fluorescence intensity ratio (ΔR) relative to the baseline ratio (R_0_) measured throughout the entire cell. We have previously reported that the dynamic range of the FRET probes used in this study is similar (Agarwal et al., 2014). To verify that our results were not affected by saturation of the probes response to cAMP, FRET responses were normalized to the magnitude of the maximal probe response observed in the same cell following exposure to saturating concentrations of the non-specific PDE inhibitor IBMX or the direct activator of AC forskolin together with isoproterenol.

The concentration of cAMP detected by each probe was estimated using the method described previously (Borner et al., 2011; Agarwal et al., 2014). Briefly, the AC inhibitor MDL12330A (100 μM) was used to define the minimum FRET response for each probe. Exposure to isoproterenol (1 μM) plus IBMX (100 μM) was used to define the maximum FRET response. This information, together with the EC_50_ for cAMP activation (Agarwal et al., 2014) was then used to estimate the actual cAMP concentration detected by each probe. The Epac2-based probes are ideally suited for studying physiologically relevant responses because their affinity for cAMP (EC_50_, 0.2 to 0.4 μM) is similar to that of its endogenous effectors (Tasken and Aandahl, 2004; Agarwal et al., 2014).

### Di-8-ANEPPS Staining

Ventricular myocytes were incubated with 5 μM Di-8-ANEPPS for 15 min at 37°C. Pluronic F-127 (0.05%) (Thermo Fisher) was included in the loading solution to aid the solubilization of the dye following manufacturer’s instructions. Cells were washed three times with extracellular solution, and dye labeled membranes were imaged using confocal microscopy.

### Methyl-β-Cyclodextrin (MβCD) Treatment

Membrane cholesterol was depleted by incubating transduced cells in the MEM-based culture medium containing 1 mM MβCD for 1 h at 37°C as previously described (Agarwal et al., 2011).

### Materials

Prostaglandin E1, MDL12330A, cilostamide, rolipram, and erythro-9-(2-hydroxy-3-nonyl) adenine hydrochloride (EHNA) were obtained from Tocris Bioscience. MEM, penicillin/streptomycin, fetal bovine serum were purchased from Life Technologies. All other reagents were purchased from Sigma-Aldrich. Isoproterenol and IBMX solutions were prepared fresh daily.

### Statistical Analysis

All data are expressed as the mean ± SEM of the indicated number of cells (*n*) isolated from *N* number of animals. Statistical significance (*p* < 0.05) was determined by Student’s *t*-test for comparison between two groups. For multiple comparisons, one -way ANOVA followed by Holm-Sidak *post hoc* test was used for normally distributed data, and Kruskal–Wallis one-way ANOVA on ranks followed by Dunn’s *post hoc* test was used for data that failed normality test. Statistical significance between two groups was defined by *p*-values of < 0.05.

## Results

The non-uniform distribution of signaling proteins between lipid raft and non-raft domains of the plasma membrane is believed to be an important factor contributing to the compartmentation of cAMP signaling in cardiac myocytes (Harvey and Calaghan, 2012). To test this hypothesis directly, we measured cAMP responses detected by three different Epac2-based biosensors: Epac2-camps is expressed uniformly throughout the cytoplasm (Nikolaev et al., 2004; Iancu et al., 2008; Agarwal et al., 2011), Epac2-MyrPalm contains an acylation sequence that targets the probe to lipid raft domains of the plasma membrane, and Epac2-CAAX contains a prenylation sequence that targets the probe to non-raft domains of the plasma membrane (Agarwal et al., 2014). Previous biochemical studies have demonstrated the effectiveness of using this strategy to target biosensors to lipid raft and non-raft membrane fractions (Zacharias et al., 2002; Depry et al., 2011; Gao et al., 2011).

Confocal images show distinctly different expression patterns for each of these probes (**Figure 1A**). As expected, Epac2-camps exhibited a diffuse cytosolic pattern, while Epac2-MyrPalm and Epac2-CAAX were targeted to the plasma membrane. However, the distribution patterns of the two membrane targeted probes were consistently different. Epac2-CAAX appeared to be evenly dispersed throughout peripheral plasma membrane as well as the membrane forming the t-tubules. On the other hand, even though Epac2-MyrPalm expression was visible in the t-tubules, it was clearly more prominent in the peripheral plasma membrane. Consistent with these observations, the ratio of the fluorescence intensity measured in the peripheral membrane relative to that measured in the interior of the cell was 0.94 ± 0.014 for Epac2-camps (*n* = 21), 1.3 ± 0.034 for Epac2-CAAX (*n* = 22), and 2.9 ± 0.19 for Epac2-MyrPalm (*n* = 10) (**Figure 1B**).

**FIGURE 2:**
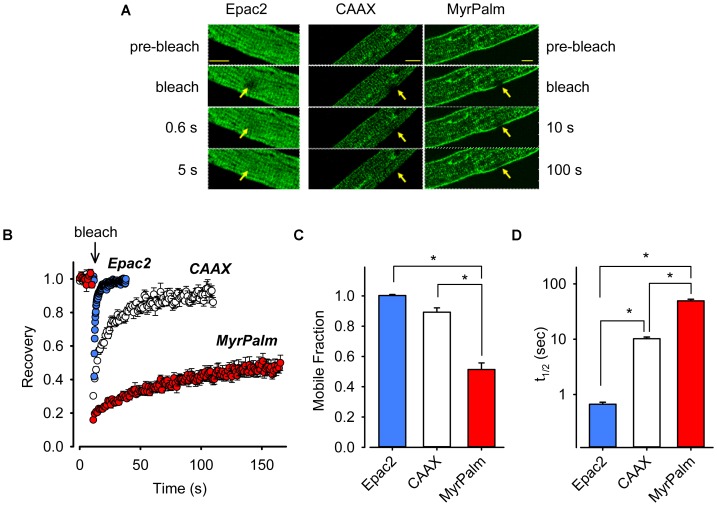
Biosensors targeted to different subcellular locations of cardiac ventricular myocytes exhibit varying mobilities. **(A)** Images from FRAP experiments in adult rat ventricular myocytes expressing Epac2-camps (Epac2, left panels), Epac2-CAAX (CAAX, middle panels), or Epac2-MyrPalm (MyrPalm, right panels). Images were taken before and at various time points after bleaching of a circular area of 5 μm in diameter (arrows). Images were captured at a greater frequency for freely diffusible Epac2 (indicated by faster recovery on the left) as compared to membrane targeted biosensors CAAX and MyrPalm (indicated by slower recovery on the right). Scale bar: 10 μm. **(B)** Time course of fluorescence recovery from photobleaching from FRAP experiments in cells expressing Epac2 (blue circles), CAAX (white circles), or MyrPalm (red circles). Summary of **(C)**, mobile fraction (M_f_), and **(D)** fluorescence recovery half-time (t_1/2_), in cells expressing Epac2-camps (n/N = 15/4, blue bars), Epac2-CAAX (n/N = 7/3, white bars) or Epac2-MyrPalm (n/N = 8/3, red bars). ^∗^*p* < 0.001, Kruskal–Wallis one-way ANOVA on Ranks followed by Dunn’s test for pairwise multiple comparisons.

Transduction with FRET biosensors required maintaining myocytes in culture for 48–72 h. To verify that the time in culture did not adversely affect the intracellular architecture of our cells, we performed experiments on both acutely isolated and cultured myocytes using the voltage sensitive membrane dye di-8-ANEPPS to monitor the integrity of sarcolemma and t-tubules (Supplementary Figure 1). The sarcolemma and the t-tubular structure appeared to be largely intact following cell culture for 72 h, consistent with previous observations (Song et al., 2008; although see Louch et al., 2004). The presence of tightly packed phospholipids and cholesterol in lipid rafts creates liquid-ordered domains that are less fluid than the surrounding plasma membrane (Pike, 2003). As a result, raft associated proteins often exhibit a mobility that differs from membrane proteins not associated with lipid rafts (Day et al., 2012). We used FRAP to measure the lateral mobility of our membrane targeted probes (**Figure 2**). We observed a near-complete recovery of Epac2-CAAX fluorescence (mobile fraction, M_f_ = 0.89 ± 0.029, *n* = 7) with a recovery haft-time (t_1/2_) of 10 ± 0.73 s. In contrast, the recovery of Epac2-MyrPalm fluorescence was significantly less complete (M_f_ = 0.51 ± 0.043) and markedly slower (t_1/2_ = 49 ± 3.4 s, *n* = 8). We then compared this to the mobility of the cytosolic Epac2-camps probe. As shown in **Figure 2**, the t_1/2_ and M_f_ for Epac2-camps was 0.66 s and 1.0 (*n* = 15), respectively. These results are consistent with the idea that while Epac2-CAAX and Epac2-MyrPalm are both targeted to the plasma membrane, they are located in distinctly different membrane domains.

Tightly-packed sphingolipids are major constituents of lipid rafts (van Meer et al., 2008). The presence of cholesterol has been proposed to render the relatively rigid arrangement of lipid rafts more fluid (Nishimura et al., 2006; van Meer et al., 2008). Therefore, to further support our conclusion that the membrane-associated FRET biosensors are correctly targeted to lipid raft and non-raft microdomains, we performed additional experiments using MβCD to deplete cholesterol from the membrane and disrupt lipid rafts (Agarwal et al., 2011). Following MβCD treatment, there was no change in either t_1/2_ or M_f_ FRAP measurements of Epac2-CAAX (Supplementary Figure 2). On the other hand, there was a small but significant increase in the t_1/2_ of Epac2-MyrPalm. This indicates that there was a selective reduction in the mobility of the lipid raft-associated biosensor in MβCD-treated cells. These results are consistent with the idea that Epac2-MyrPalm and Epac2-CAAX were being targeted to different membrane domains (Agarwal et al., 2014).

**FIGURE 3:**
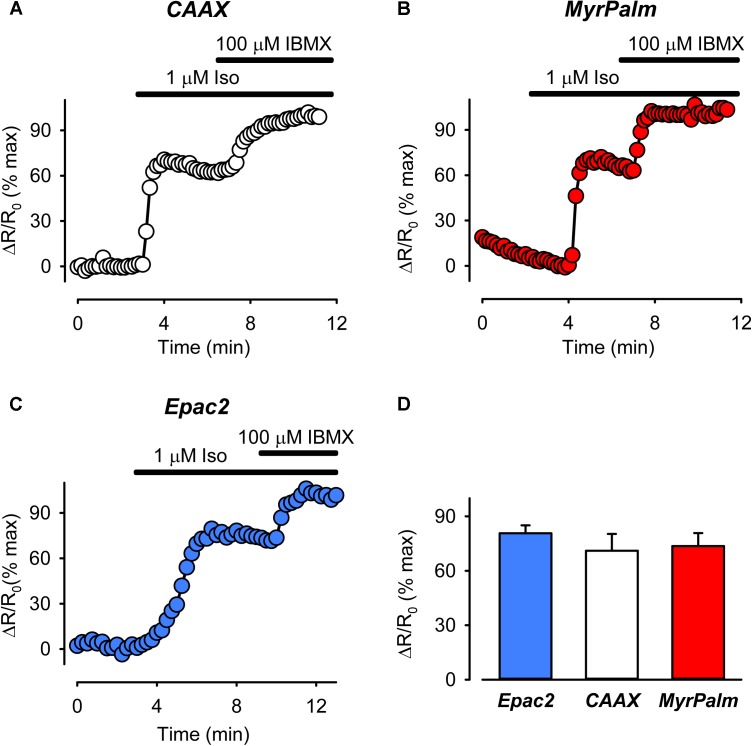
The magnitude of cAMP responses detected by biosensors targeted to different subcellular locations are similar following treatment with the βAR agonist isoproterenol (Iso). **(A–C)** Representative time course of changes in the normalized FRET response (ΔR/R_0_) in cells expressing Epac2-CAAX (CAAX, white circles), Epac2-MyrPalm (MyrPalm, red circles), and Epac2-camps (Epac2, blue circles), under control conditions, and following exposure to 1 μM Iso, and 1 μM Iso plus 100 μM IBMX. **(D)** Summary of average FRET responses to 1 μM Iso in cells expressing Epac2-camps (n/N = 8/6, blue bar), Epac2-CAAX (n/N = 10/5, white bar), and Epac2-MyrPalm (n/N = 14/5, red bar). Responses to 1 μM Iso were not significantly different between the probes (one-way ANOVA).

Next we compared cAMP responses detected by the different probes following receptor activation. In cardiac myocytes, βARs are found in lipid raft and non-raft fractions of the plasma membrane, while EPRs are only found in non-raft fractions (Rybin et al., 2000; Ostrom et al., 2001, 2002; Head et al., 2005; Agarwal et al., 2011). We asked if it was possible to observe differences in the cAMP responses produced by stimulation of βARs and EPRs. As shown in **Figure 3**, all three probes detected robust, but non-saturating, increases in cAMP activity following activation of βARs with isoproterenol. The magnitude of the normalized FRET responses were not significantly different in cells expressing Epac2-camps (81 ± 4.4%, *n* = 8), Epac2-CAAX (71 ± 9.3%, *n* = 10), and Epac2-MyrPalm (74 ± 7.2, *n* = 14). These results are consistent with the idea that βARs stimulate cAMP production in subcellular locations associated with lipid raft as well as non-raft domains of the plasma membrane.

Previous studies have demonstrated that cholesterol depletion preferentially alters cAMP signaling associated with lipid rafts in cardiac myocytes (Rybin et al., 2000; Agarwal et al., 2011; Macdougall et al., 2012; Pugh et al., 2014). Therefore, we reasoned that, following MβCD treatment, we should only detect changes in cAMP responses detected by biosensors targeted to lipid raft domains. Consistent with this idea, in MβCD-treated cells exposed to a submaximally stimulating concentration of isoproterenol (3 nM) Epac2-camps and Epac2-CAAX produced FRET responses that were not significantly different from control cells. However, Epac2-MyrPalm responses were significantly smaller (Supplementary Figures 3A–C). Previous studies have actually found that cholesterol depletion enhances cAMP production by receptors found in caveolae/lipid rafts. The reason for the decrease observed here is likely due to a change in the function of the raft-targeted biosensor itself. Consistent with this idea, cholesterol depletion also selectively reduced the ability of Epac2-MyrPalm to respond to maximal stimulation with isoproterenol plus IBMX (Supplementary Figure 3D). These results suggest that lipid raft disruption selectively alters the ability of the Epac2-MyrPalm probe to respond to changes in cAMP. They also further support the conclusion that Epac2-MyrPalm and Epac2-CAAX are targeted to different domains in the plasma membrane.

**FIGURE 4:**
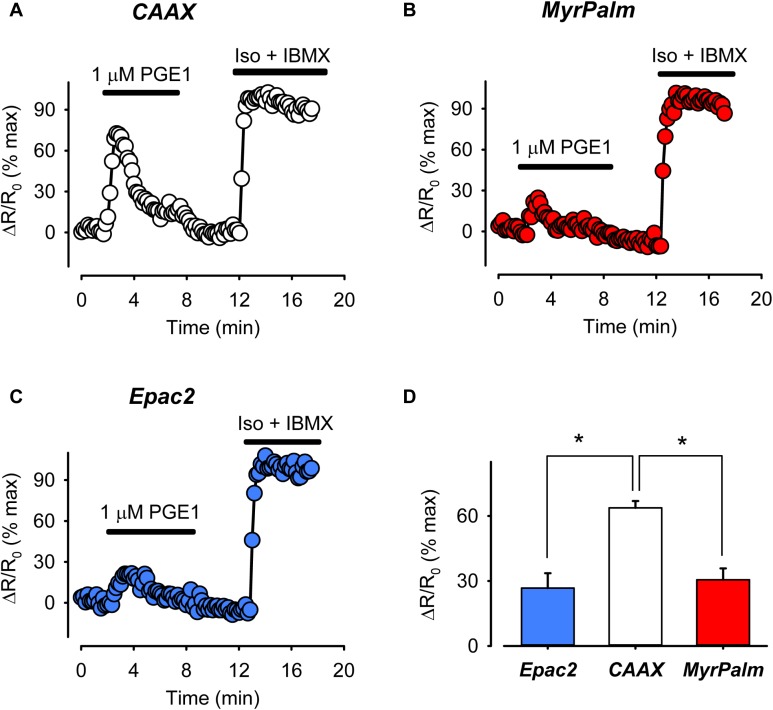
The prostaglandin receptor (EPR) agonist PGE1 produces greater cAMP response in non-raft-associated membrane domains. **(A–C)** Representative time course of changes in the normalized FRET response (ΔR/R_0_) in cells expressing Epac2-CAAX (CAAX, white circles), Epac2-MyrPalm (MyrPalm, red circles), and Epac2-camps (Epac2, blue circles), under control conditions and following exposure to 1 μM PGE1. Maximal response was elicited by subsequent exposure to 1 μM Iso plus 100 μM IBMX (Iso + IBMX). **(D)** Summary of average FRET responses to 1 μM PGE1 in cells expressing Epac2-camps (n/N = 5/3, blue bar), Epac2-CAAX (n/N = 5/3, white bar), and Epac2-MyrPalm (n/N = 6/3, red bar). Epac2-CAAX response to 1 μM PGE1 was significantly different from Epac2-camps and Epac2-MyrPalm responses (^∗^*p* < 0.05, one-way ANOVA followed by Holm-Sidak method for pairwise multiple comparisons).

In the next set of experiments, we compared FRET responses produced by EPR activation using 1 μM PGE1. Although changes in cAMP activity could be sensed by all three probes, the peak FRET response detected by Epac2-CAAX was 64 ± 3.2% (*n* = 5) of maximal. This was significantly greater than the magnitude of the responses detected by either Epac2-MyrPalm (31 ± 5.2%, *n* = 6) or Epac2-camps (27 ± 6.9%, *n* = 5) (**Figure 4**). These results are consistent with the idea that EPRs preferentially stimulate cAMP production in subcellular locations associated with non-raft domains of the plasma membrane.

**FIGURE 5:**
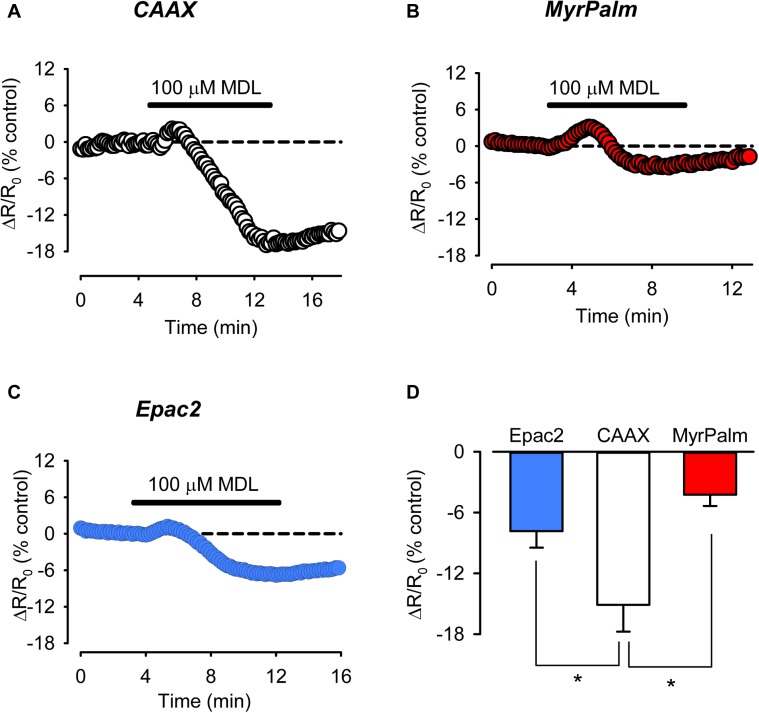
Inhibition of basal AC activity leads to greater reduction of cAMP activity in non-raft associated membrane domains. **(A–C)** Representative time course of changes in the FRET response (ΔR/R_0_) in cells expressing Epac2-CAAX (CAAX, white circles), Epac2-MyrPalm (MyrPalm, red circles), and Epac2-camps (Epac2, blue circles), under control conditions and following exposure to the AC inhibitor MDL12330A (MDL; 100 μM). **(D)** Summary of average FRET responses to 100 μM MDL in cells expressing Epac2-camps (n/N = 11/5, blue bar), Epac2-CAAX (n/N = 10/6, white bar), and Epac2-MyrPalm (n/N = 7/3, red bar). Epac2-CAAX response to 100 μM MDL was significantly different from Epac2-camps and Epac2-MyrPalm responses (^∗^*p* < 0.05, Kruskal–Wallis one-way ANOVA on Ranks followed by Dunn’s test for pairwise multiple comparisons). Reference line (dotted) at zero has been added for clarity.

It is often assumed that under basal conditions cAMP concentrations are uniformly low throughout the cell and that it is only in response to agonist stimulation that levels increase enough to activate FRET based biosensors. However, it has previously been demonstrated that in adult ventricular myocytes, basal levels of cAMP are actually high enough to partially activate the Epac2-camps biosensor (Iancu et al., 2008; Borner et al., 2011). This indicates that basal levels of cAMP production are high enough to maintain a significant level of cAMP activity throughout the bulk cytoplasmic compartment of these cells. Consistent with this idea, we found that exposure to 100 μM MDL12330A, an irreversible inhibitor of AC activity, produced a decrease in the FRET response of the Epac2-camps probe (**Figure 5C**). We found that exposure to MDL also inhibited the baseline response of the Epac2-MyrPalm and Epac2-CAAX probes (**Figures 5A,B**). Therefore, it appears that cAMP levels are constitutively elevated in subcellular domains associated with the plasma membrane, as well. However, the inhibitory effect of MDL had its greatest effect on cAMP responses detected by Epac2-CAAX (**Figure 5D**).

**FIGURE 6:**
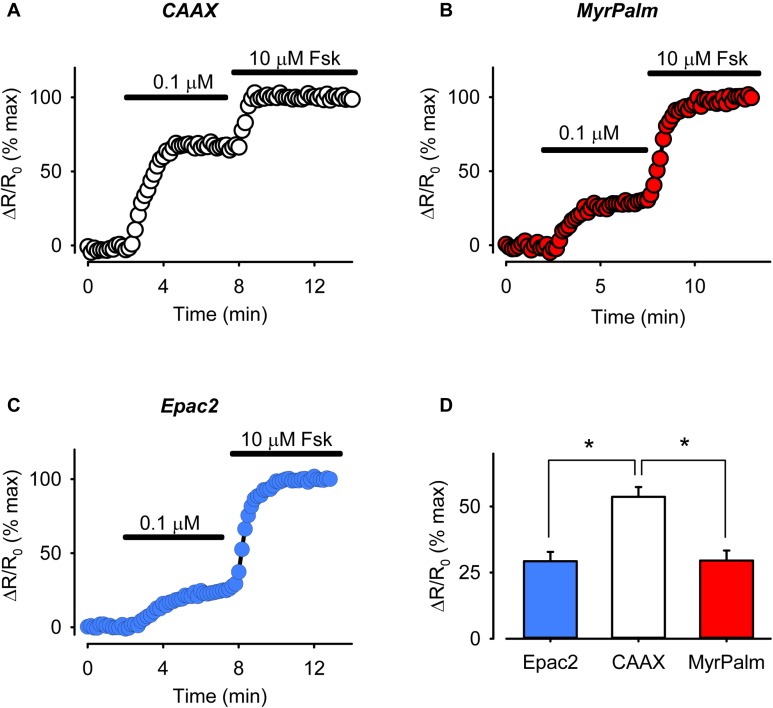
The magnitude of cAMP responses detected in non-raft membrane domains is higher following direct AC stimulation. **(A–C)** Representative time course of changes in magnitude of relative FRET response (ΔR/R_0_) in cells expressing Epac2-CAAX (CAAX, white circles), Epac2-MyrPalm (MyrPalm, red circles) and Epac2-camps (Epac2, blue circles), under control conditions and following exposure to sub-maximally (0.1 μM) and maximally (10 μM) stimulating concentration of the AC activator forskolin (Fsk). **(D)** Summary of average FRET responses to 0.1 μM Fsk in cells expressing Epac2-camps (n/N = 8/3, blue bar), Epac2-CAAX (n/N = 8/3, white bar), and Epac2-MyrPalm (n/N = 8/3, red bar). Epac2-CAAX response to 0.1 μM Fsk was significantly different from Epac2-camps and Epac2-MyrPalm responses (^∗^*p* < 0.001, one-way ANOVA followed by Holm-Sidak method for pairwise multiple comparisons).

The fact that there is a detectable amount of cAMP present under basal conditions indicates that there must be significant AC activity even in the absence of receptor activation. Likewise, non-uniform distribution of basal AC activity may then be one possible explanation for the differences in cAMP activity detected in different subcellular locations, especially those near the membrane. To determine if this might be the case, we compared the sensitivity of the responses detected by each probe to forskolin, a direct activator of AC (**Figure 6**). The size of the response produced by a submaximally stimulating concentration of forskolin should correlate with amount of basal AC activity responsible for producing cAMP in that location (Agarwal et al., 2014). In cells expressing Epac2-CAAX, 0.1 μM forskolin produced a FRET response that was 54 ± 3.7% of the maximal response (*n* = 8) elicited by 10 μM forskolin. This was significantly greater than the size of the response that this submaximally stimulating concentration of forskolin produced in cells expressing either Epac2-camps (29 ± 3.6%, *n* = 8) or Epac2-MyrPalm (30 ± 3.8%, *n* = 8). These data are consistent with the hypothesis that there is a greater level of basal AC activity associated with non-raft domains of the plasma membrane. In agreement with this conclusion, we also saw a greater reduction in cAMP responses in non-raft domains as compared to lipid rafts or bulk cytosolic regions, following inhibition of AC activity using submaximally inhibiting concentration (30 μM) of MDL (Supplementary Figure 4).

**FIGURE 7:**
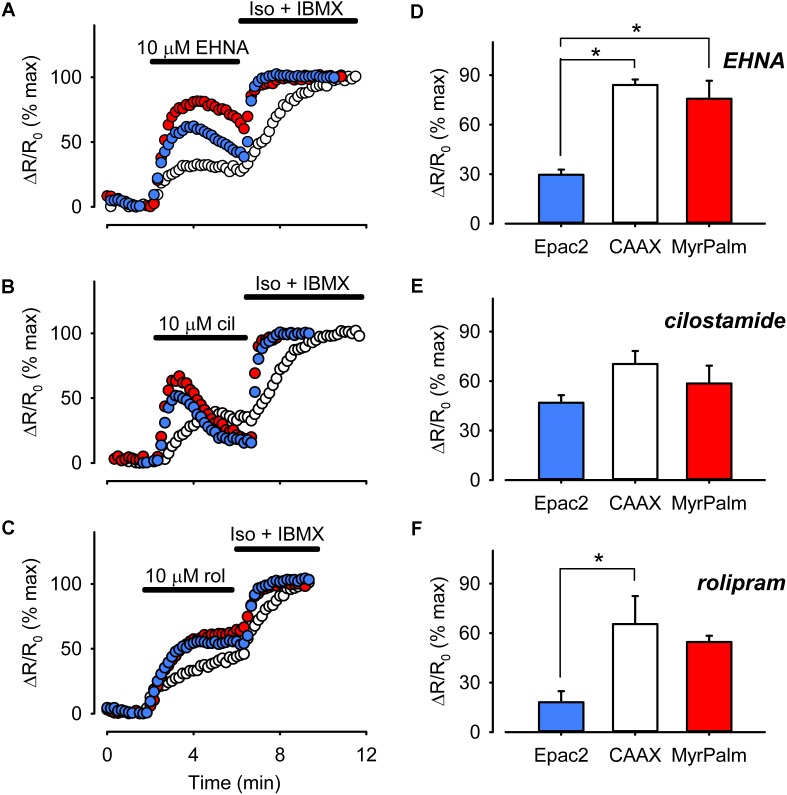
Differential PDE activity is associated with different microdomains. **(A–C)** Representative time course of changes in the magnitude of the normalized FRET response (ΔR/R_0_) recorded in cells expressing Epac2-camps (Epac2, blue circles), Epac2-CAAX (CAAX, white circles), and Epac2-MyrPalm (MyrPalm, red circles) under control conditions and following exposure to the selective PDE2 inhibitor EHNA **(A)**, the selective PDE3 inhibitor cilostamide **(B)**, or the selective PDE4 inhibitor rolipram **(C)**. The maximal response was elicited by subsequent exposure to 1 μM Iso plus 100 μM IBMX (Iso + IBMX). **(D–F)** Comparison of average changes in normalized FRET responses detected by Epac2-camps (blue bars), Epac2-CAAX (white bars), and Epac2-MyrPalm (red bars) following treatment with 10 μM EHNA (n/N = 5/3, Epac2; 5/3, CAAX; 5/3, MyrPalm; **D**), 10 μM cilostamide (n/N = 6/4, Epac2; 7/3, CAAX; 6/3, MyrPalm; **E**), or 10 μM rolipram (n/N = 6/4, Epac2; 5/3, CAAX; 6/3, MyrPalm; **F**) (^∗^*p* < 0.05, Kruskal–Wallis one-way ANOVA on Ranks followed by Dunn’s test for pairwise multiple comparisons).

Differences in basal cAMP levels might also be explained by differences in PDE activity. Targeted expression of different PDE isoforms is involved in compartmentalizing cAMP signaling in various cell types (Conti et al., 2014). In mammalian ventricular myocytes, PDE2, PDE3, and PDE4 play critical roles in regulating cAMP levels (Mika et al., 2012). To determine the relative contribution of each of these PDE isoforms to cAMP responses associated with different subcellular locations, we monitored the effects of selectively inhibiting PDE2 with 10 μM EHNA, PDE3 with 10 μM cilostamide, or PDE4 with 10 μM rolipram (Di Benedetto et al., 2008). As shown in **Figure 7**, inhibition of all three isoforms produced responses that could be detected by each of the probes.

Inhibition of PDE3 activity produced peak FRET responses that were 47 ± 4.6% (*n* = 6), 70 ± 7.9% (*n* = 7), and 59 ± 11% (*n* = 6) of maximal in bulk cytosolic, non-raft, and lipid raft domains, respectively. These were not significantly different from one another. However, inhibition of PDE2 and PDE4 had greater effects on cAMP responses detected by Epac2-CAAX and Epac2-MyrPalm than they had on the responses detected by Epac2-camps. Inhibition of PDE2 activity produced peak responses that were 30 ± 3.1% (*n* = 5), 84 ± 3.4% (*n* = 5), and 76 ± 11% (*n* = 5) of maximal in the bulk cytosolic, non-raft, and lipid raft domains, respectively. Inhibition of PDE4 activity produced FRET responses that were 18 ± 6.7% (*n* = 6), 69 ± 14% (*n* = 5), and 55 ± 3.7% (*n* = 6) in the same corresponding locations. These data indicate that all three PDE isoforms play an important role in regulating cAMP activity near the plasma membrane, with no difference in the relative contribution to lipid raft and non-raft domains. However, PDE3 appeared to contribute more than the other PDE isoforms in regulating cAMP in the bulk cytoplasmic domain.

## Discussion

One goal of the present study was to determine if it is possible to detect differences in cAMP levels in subcellular locations associated with lipid raft and non-raft domains of the plasma membrane in adult cardiac myocytes using versions of the Epac2-camps biosensor targeted to those locations (Agarwal et al., 2014). We found that Epac2-CAAX was expressed uniformly throughout the plasma membrane, including the membrane lining the t-tubules. However, Epac2-MyrPalm was concentrated more in the peripheral plasma membrane (see **Figure 1**). The expression pattern of Epac2-MyrPalm is similar to that of caveolin-3, the primary caveolin found in cardiac myocytes (Head et al., 2005; Balijepalli et al., 2006). However, this should not be taken to mean that Epac2-MyrPalm is targeted specifically to caveolae, since caveolins represent only a subset of lipid rafts. Furthermore, it has been suggested that caveolin itself may be associated with lipid rafts that do not form caveolae (Head et al., 2005; Nichols et al., 2010). Results from FRAP experiments further support the conclusion that the biosensors used in this study are expressed in distinctly different locations in myocytes (**Figure 2**). Not only was the fluorescence recovery of Epac2-MyrPalm much slower than that of Epac2-CAAX, but lipid raft disruption selectively altered recovery of the MyrPalm probe. Disrupting lipid rafts also selectively affected the ability of the Epac2-MyrPalm to detect cAMP responses to βAR stimulation (Supplementary Figure 3).

It has been reported that in adult ventricular myocytes, βARs are found in lipid raft and non-raft fractions of the plasma membrane, while EPRs are found exclusively in non-raft fractions (Rybin et al., 2000; Ostrom et al., 2001, 2002; Head et al., 2005; Agarwal et al., 2011). Furthermore, we previously demonstrated that disrupting lipid rafts selectively alters βAR production of cAMP in type II PKA signaling domains, without affecting global cAMP responses to βAR stimulation or cAMP response to EPR stimulation (Agarwal et al., 2011). This suggests that receptors associated with lipid raft domains of the plasma membrane can produce localized changes in cAMP that do not contribute to global responses. In the present study, we directly tested this hypothesis by comparing changes in cAMP activity detected by Epac2-MyrPalm and Epac2-CAAX.

There were no differences in the magnitude of the FRET responses detected by any of the probes used in the present study following activation of βARs with submaximally and maximally stimulating concentration of isoproterenol (see **Figure 3** and Supplementary Figure 3). However, interpretation of these results could be complicated by the fact that we found evidence for differences in basal cAMP (**Figure 5**), which might mean that there were actual differences in concentration of cAMP produced following receptor activation (Agarwal et al., 2014). Using the method described by Borner et al. (2011), we estimated the actual cAMP concentration detected under different conditions. In response to 3 nM Iso, the concentrations of cAMP detected by Epac2-camps (711 ± 74.1 nM), Epac2-CAAX (636 ± 79.9 nM), and Epac2-MyrPalm (688 ± 104 nM) were not statistically different from each other. Whether or not the same is true following exposure to 1 μM Iso is less certain, since the responses were too close to saturating the probes to make reliable estimates of the cAMP concentration (Iancu et al., 2008; Agarwal et al., 2014). However, our results support the idea that βAR stimulation in general produces uniform responses throughout the cell. Future studies with these probes, could be useful in defining microdomain specific cAMP responses produced by selective activation of the β_1_ and β_2_AR subtypes.

In contrast to the effects of isoproterenol, we found that activation of EPRs with PGE1 produced a distinctly different pattern of cAMP responses (see **Figure 4**). Consistent with our previous finding, PGE1 produced a transient increase in cAMP that was detected in the bulk cytosolic compartment of adult ventricular myocytes by Epac2-camps (Warrier et al., 2007; Agarwal et al., 2011). PGE1 also produced a response detected by both the MyrPalm and CAAX probes. However, the estimated peak concentration of cAMP detected by Epac2-CAAX (840 ± 79.2 nM) was more than twice that detected by Epac2-camps (332 ± 77.0 nM) or Epac2-MyrPalm (405 ± 67.5 nM). Feedback activation of PDE4 by PKA-mediated phosphorylation and desensitization of EPRs may be involved in generating transient cAMP responses following treatment with PGE1 (Rich et al., 2007). These results demonstrate that it is possible to directly measure differences in cAMP responses associated with distinct microdomains of the plasma membrane. It also suggests that cAMP produced by EPRs in non-raft regions of the plasma membrane is limited in its ability to reach other signaling domains throughout the cell. Previous studies have suggested that PDE3 and PDE4 activity contribute to this behavior (Rochais et al., 2006) but other factors are likely to be involved as well (Saucerman et al., 2014).

We also determined that it was possible to detect differences in basal cAMP activity associated with the different membrane domains (see **Figure 5**). It has previously been determined that basal cAMP levels in the bulk cytoplasmic compartment of adult ventricular myocytes are elevated, even in the absence of receptor activation (Iancu et al., 2008; Borner et al., 2011), but it was unclear if the same is true for cAMP levels near the plasma membrane. Consistent with earlier studies, we found that inhibition of basal AC activity with MDL caused a significant decrease in cAMP activity detected in the bulk cytoplasmic compartment. From these results, we estimated the cAMP concentration at 152 ± 34.7 nM (*n* = 11). This is lower than previous estimates of ∼1.2 μM cAMP found in bulk cytoplasmic compartment of adult guinea pig and mouse ventricular myocytes (Iancu et al., 2008; Borner et al., 2011), suggesting that there may be species dependent differences. It should be noted, however, that the EC_50_ value for the Epac2-camps probe used to calculate basal cAMP levels in this study is slightly lower than that described in previous reports (Nikolaev et al., 2004; Iancu et al., 2008), which could have also contributed to the reported differences in basal cAMP concentration.

Exposure to MDL also produced a decrease in cAMP activity detected by both the MyrPalm and CAAX probes. The results indicate that the basal level of cAMP associated with lipid raft domains 127 ± 37.5 nM (*n* = 7) is similar to that found in the bulk cytoplasmic compartment. This is also similar to previous estimates of the basal cAMP concentration existing in type II PKA signaling domains (Iancu et al., 2007). However, the concentration of cAMP associated with non-raft domains of the plasma membrane was found to be significantly higher (274 ± 41.6 nM, *n* = 10). This pattern is similar to what we previously found in HEK293 cells, where basal levels of cAMP associated with non-raft regions of the plasma membrane were higher than those found elsewhere throughout the cell (Agarwal et al., 2014).

The results of the present study suggest that differences in AC activity contribute to the dissimilarities in basal cAMP activity associated with the various membrane domains. This was supported by differences in the sensitivity of the cAMP responses detected by Epac2-CAAX and Eapc2-MyrPalm following exposure to a submaximally stimulating concentration of forskolin (see **Figure 6**). Possible explanations for these differences could involve mechanisms known to regulate AC activity. For example, it has previously been shown that ACs are inhibited by direct interactions with the scaffolding domain of caveolins, a component of caveolae (Toya et al., 1998), which are a subset of lipid rafts. This can explain why disrupting lipid rafts has been reported to enhance cAMP production in these cells (Rybin et al., 2000; Head et al., 2005; Agarwal et al., 2011). In addition, AC activity can also be inhibited by PKA-dependent phosphorylation (Iwami et al., 1995), and type II PKA is also associated with caveolar fractions of the plasma membrane (Rybin et al., 2000; Balijepalli et al., 2006; Nichols et al., 2010). These factors could explain the lower basal AC activity associated with lipid raft domains. It is also interesting to speculate about which AC isoforms are involved. Adenylyl cyclase types 5 and 6 are the predominant isoforms found in cardiac myocytes. They are also often associated specifically with caveolae (Rybin et al., 2000; Head et al., 2005; Balijepalli et al., 2006). However, there is also evidence that cardiac myocytes express AC types 4 and 7 (Defer et al., 2000), which are typically found in non-raft fractions of the plasma membrane (Ostrom and Insel, 2004; Cooper, 2005).

Variations in basal cAMP levels could also be explained by differences in PDE activity. However, we did not find any evidence that PDE2, PDE3, or PDE4 plays a role in explaining any of our observations (see **Figure 7**). We did find that PDE2 and PDE4 have a greater effect on cAMP activity near the plasma membrane than they do in the bulk cytoplasmic compartment. This is consistent with biochemical studies demonstrating that PDE2 and PDE4 are predominantly associated with membrane fractions of cardiac myocytes, whereas PDE3 is found in both cytosolic and membrane fractions (Mika et al., 2012). Yet this does not explain why basal cAMP levels near the plasma membrane are essentially the same or even higher than they are in the bulk cytoplasmic compartment. Furthermore, we found evidence that PDE2, PDE3, and PDE4 appear to be equally important in regulating cAMP activity associated with lipid raft and non-raft domains of the plasma membrane. There was no evidence that any of these three PDE isoforms plays a role in explaining the differences in basal cAMP levels associated with these membrane domains. Although cardiac myocytes also express Ca^2+^-dependent PDE1 activity, it is not believed to contribute significantly to the regulation of cAMP responses in adult rat ventricular myocytes (Verde et al., 1999; Richter et al., 2011), especially in non-contracting myocytes (Sprenger et al., 2016).

It is interesting to note the transient nature of cAMP responses following inhibition of certain PDE isoforms. This was true for some responses detected near the plasma membrane, but not those in the bulk cytoplasmic compartment (see **Figure 7**). It was most noticeable following inhibition of PDE3, but also to some extent with inhibition of PDE2. One possible explanation for this observation is that an increase in cAMP near the membrane is activating PKA, which can then phosphorylate PDE4, increasing its activity in a negative feedback manner (Rochais et al., 2004). This would explain the absence of transient responses following inhibition of PDE4. These results suggest that there is significant interaction between different PDE isoforms near the plasma membrane (Zhao et al., 2016).

Earlier studies looked specifically at cAMP responses near the plasma membrane in adult ventricular myocytes using the current generated by exogenous cyclic nucleotide gated (CNG) ion channels as a reporter. However, that probe was unable to detect any change in cAMP activity following EPR activation alone, or following inhibition of either PDE3 or PDE4 activity alone. CNG channels also fail to respond to changes in cAMP produced by exposure to IBMX alone, even though this stimulus maximally activates other cAMP responses in adult ventricular myocytes (Rochais et al., 2004, 2006). Responses detected by CNG channels and Epac2-based FRET probes also differ in HEK293 cells (Rich et al., 2007; Agarwal et al., 2014). In HEK293 cells, CNG-channels detect a transient response to PGE1, while we previously reported that our FRET based biosensors detect a sustained response (Agarwal et al., 2014). The reasons for these differences are not completely clear. It should be noted that, unlike the FRET probes used in the present study, the membrane domain in which CNG channels are expressed is not well-characterized.

Another earlier study compared the responses detected by cytosolic and plasma membrane targeted Epac2-based biosensors in neonatal myocytes and found that PDE4B activity regulates subsarcolemmal cAMP activity, but not cAMP activity in the cytosolic compartment (Mika et al., 2014). While this is slightly different from what we found, it is difficult to compare those results with the results of the present study because the exact nature of the biosensors used was not reported. Furthermore, our experiments were conducted using adult myocytes, and we did not investigate the role of individual splice variants of the different PDE isoforms.

Even though cAMP compartmentation is common to all cells, the present study highlights the importance of the specific cell type used when characterizing the phenomenon and its underlying mechanisms. The results obtained from studies using the same three probes in HEK 293 and airway smooth muscle (HASM) cells are quite different from those found in the present study (Agarwal et al., 2014, 2017). In all three cell types, prostaglandins stimulate cAMP production by activating EPRs found in non-raft domains. However, the ability of the cAMP produced by those receptors to reach other subcellular locations varies by cell type. We found little evidence for compartmentation of the EPR response in HEK cells, while the EPR production of cAMP appears to be progressively more restricted in cardiac myocytes and HASM cells. There are also major differences in basal cAMP levels in the different cell types. No significant amount of basal cAMP could be detected in HASM cells, unlike HEK cells and cardiac myocytes, where basal levels are significantly elevated and vary between different subcellular locations. The differences in basal cAMP also reflect significant differences in basal AC and/or PDE activity. All three cell types demonstrate evidence for greater AC activity in non-raft domains. However, the absolute level of AC activity appears to be greatest in cardiac myocytes. This is demonstrated by differences in the sensitivity to PDE inhibition. In HEK and HASM cells, IBMX alone has little or no effect on cAMP anywhere, while IBMX alone produces saturating responses everywhere in cardiac myocytes. It is only with selective PDE inhibitors that we were able to measure non-saturating responses.

## Conclusion

In the present study, we demonstrate that it is possible to directly measure differences in basal and receptor stimulated cAMP activity associated with lipid raft and non-raft domains of the plasma membrane in adult cardiac ventricular myocytes. This means that when using biosensors targeted to the plasma membrane to study cAMP signaling in subsarcolemmal spaces, it is important to consider the specific microdomain in which the probes are expressed.

The ability of cardiac myocytes to generate localized pools of cAMP within different subcellular microdomains suggests the presence of mechanisms that prevent uniform changes in cAMP levels throughout the cell. Many previous studies have focused on heterogeneities in PDE activity. In the present study, we demonstrate that heterogeneities in membrane receptor distribution as well as heterogeneities in basal AC activity associated with lipid raft and non-raft domains of the plasma membrane also play an important role.

Our results suggest that cAMP produced by at least some receptors found outside of lipid rafts is limited in its ability to diffuse throughout the cell. This correlates with the inability of cAMP produced by EPRs to elicit changes in the electrical or mechanical activity of cardiac myocytes (Warrier et al., 2007; Agarwal et al., 2011), despite that it can elicit cardioprotective effects (Xiao et al., 2004). What actually limits the movement of this cAMP is still unknown. We have previously demonstrated that it is unlikely to involve PDE activity alone (Warrier et al., 2007). More recent studies have identified spatially-restricted spaces along with slow diffusion of cAMP due to PKA buffering as other possible contributing factors (Feinstein et al., 2012; Saucerman et al., 2014; Agarwal et al., 2016; Richards et al., 2016; Yang et al., 2016). How the interplay between these various elements affects physiologic and pathologic responses remains to be fully explored.

## Author Contributions

SA, CC, and RH conceived and designed the experiments. SA, JG, and MC performed the experiments and analyzed the data. SA, P-CY, CC, and RH interpreted the data. SA and RH wrote the manuscript.

## Conflict of Interest Statement

The authors declare that the research was conducted in the absence of any commercial or financial relationships that could be construed as a potential conflict of interest.

## Abbreviations

AC: adenylyl cyclase
AKAP: A kinase anchoring protein
βAR: β-adrenergic receptor
cAMP: 3′,5′-cyclic adenosine monophosphate
Cil: cilostamide
eCFP: enhanced cyan fluorescent protein
EHNA: erythro-9-(2-hydroxy-3-nonyl) adenine hydrochloride
EPR: E-type prostaglandin receptor
Epac2-camps: exchange protein activated by cAMP type 2 based cAMP biosensor
Epac2-MyrPalm: Epac2-camps biosensor with an acylation targeting sequence
Epac2-CAAX: Epac2-camps biosensor with a prenylation targeting sequence
eYFP: enhanced yellow fluorescent protein
FRAP: fluorescence recovery after photobleaching
FRET: fluorescence resonance energy transfer
Fsk: forskolin
GPCR: G protein-coupled receptor
IBMX: 3-isobutyl-1-methylxanthine
Iso: isoproterenol
MβCD: methyl-β-cyclodextrin
MDL: MDL12330A
PDE: phosphodiesterase
PKA: protein kinase A
PGE1: prostaglandin E1
Rol: rolipram.
