# Supplementary material for: Compartmentalized cAMP Signaling Associated With Lipid Raft and Non-raft Membrane Domains in Adult Ventricular Myocytes
Source: Front Pharmacol. 2018 Apr 23;9:332. doi: 10.3389/fphar.2018.00332 (PMC5925456; doi:10.3389/fphar.2018.00332)
Supplement: Supplementary file 1 [file Data_Sheet_1.pdf]

*Supplementary Material*

# Compartmentalized cAMP Signaling Associated with Lipid Raft and Non-Raft Membrane Domains in Adult Ventricular Myocytes

**Shailesh R. Agarwal*, Jackson Gratwohl, Mia Cozad, Pei-Chi Yang, Colleen E. Clancy, and Robert D. Harvey**

*** Correspondence:** Corresponding Author: sagarwal@unr.edu

## 1. Supplementary Figures

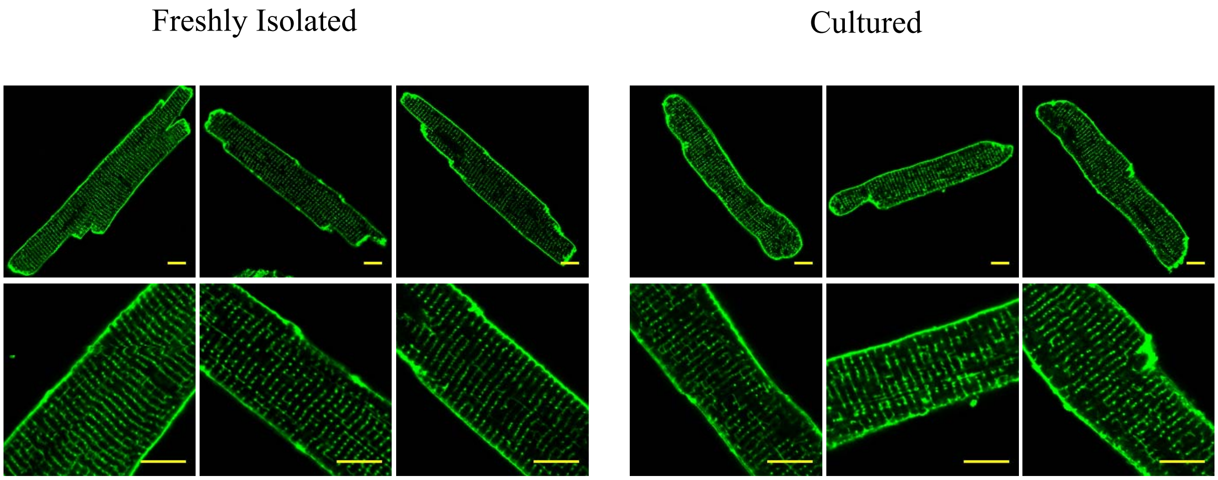


**Supplementary Figure 1. Intracellular morphology of cardiac myocytes is largely preserved following tissue culture.** Cardiac myocytes were isolated from adult rats and cultured in 6 well plates for 72 hours. Cells were stained with di-8-ANEPPS and imaged on confocal microscope. Images were acquired of cells on day 0 (freshly isolated, left panels, n/N = 29/3) and after 72 hours in culture (cultured, right panels, n/N = 37/3) using 60X, 1.42 NA objective at 2X (upper panels) and 5X (lower panels) zoom. Images shown are representative examples from three separate cell isolations for both freshly isolated and cultured myocytes. Scale bar: 10 µm.

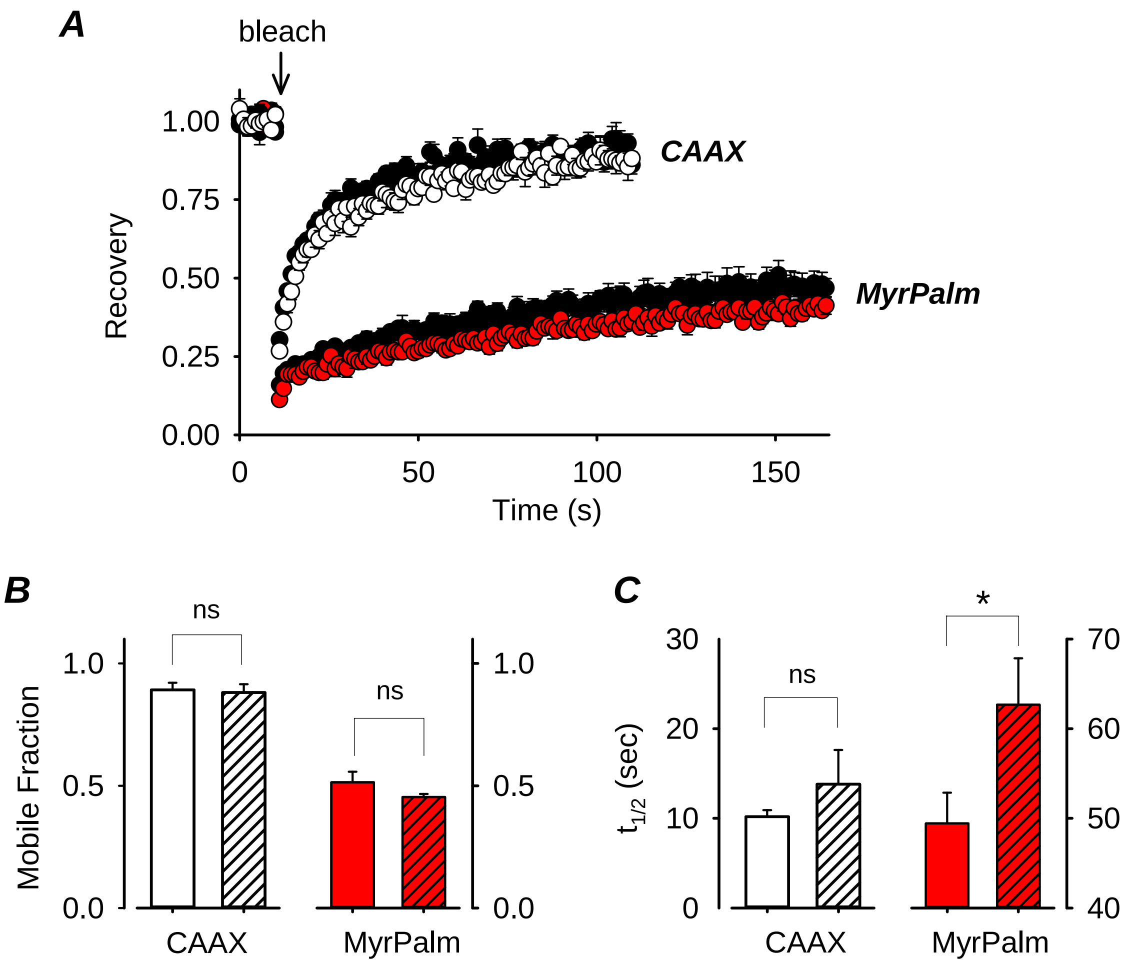


**Supplementary Figure 2. Cholesterol depletion preferentially alters mobility of Epac2-MyrPalm, but not Epac2-CAAX. A,** Time course of fluorescence recovery after photobleaching (FRAP) in control and MβCD-treated ventricular myocytes expressing Epac2-CAAX (CAAX, black circles, control; white circles, MβCD-treated) or Epac2-MyrPalm (MyrPalm, control, black circles; MβCD-treated, red circles). Summary of **(B),** mobile fraction, and **(C),** fluorescence recovery half-time ($t_{1/2}$) in control and MβCD-treated cells expressing CAAX (control, n/N = 7/3, white solid bars; MβCD, n/N = 5/3, white hatched bars) or MyrPalm (control, n/N = 8/3, red solid bars; MβCD, n/N = 5/2, red hatched bars). * $p < 0.05$ (two-tailed Student's t-test), ns = not significant. The data for control cells are same as those shown in figure 2 of the manuscript. These data are included to compare with the fluorescence recovery after cholesterol depletion.

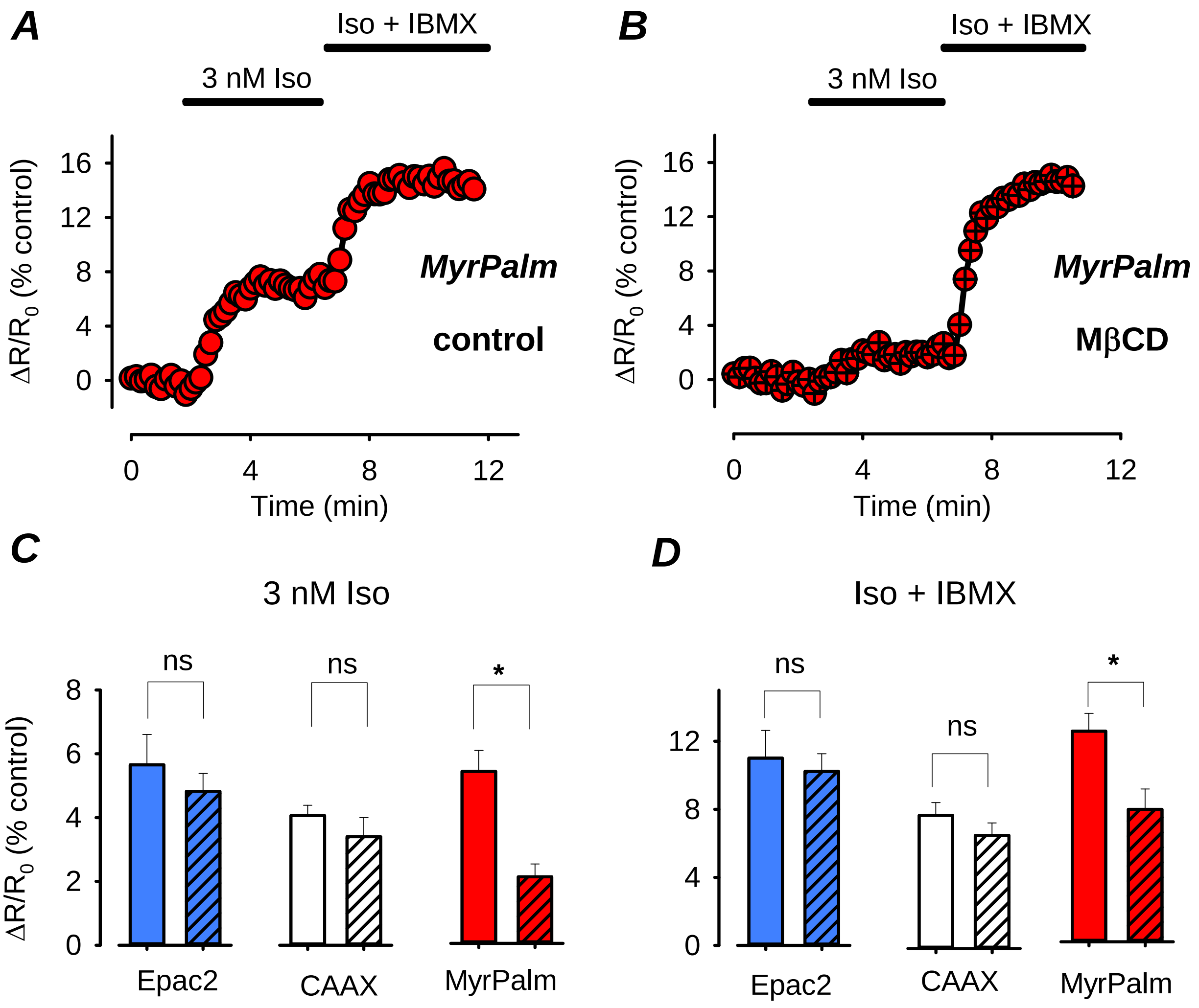


**Supplementary Figure 3. Cholesterol depletion preferentially alters cAMP responses in Epac2-MyrPalm-, but not Epac2-camps-, or Epac2-CAAX-expressing myocytes.** Representative time course of changes in FRET response ($\Delta R/R_0$) in myocytes expressing Epac2-MyrPalm (MyrPalm) in control (**A**, red solid circles) and MβCD-treated (**B**, red crossed circles) cells following treatment with 3 nM Iso and 1 µM Iso plus 100 µM IBMX. Summary of average FRET responses to 3 nM Iso (**C**), and 1 µM Iso plus 100 µM IBMX (**D**) in control and MβCD-treated cells expressing Epac2 ( n/N = 6/3, control, blue solid bars; 6/3, MβCD, blue hatched bars), CAAX (n/N = 9/3, control, white solid bars; 7/4, MβCD, white hatched bars), and MyrPalm ( n/N = 8/4, control, red solid bars; 15/6, MβCD, red hatched bars). * $p < 0.001$ (two-tailed Student's t-test), ns = not significant.

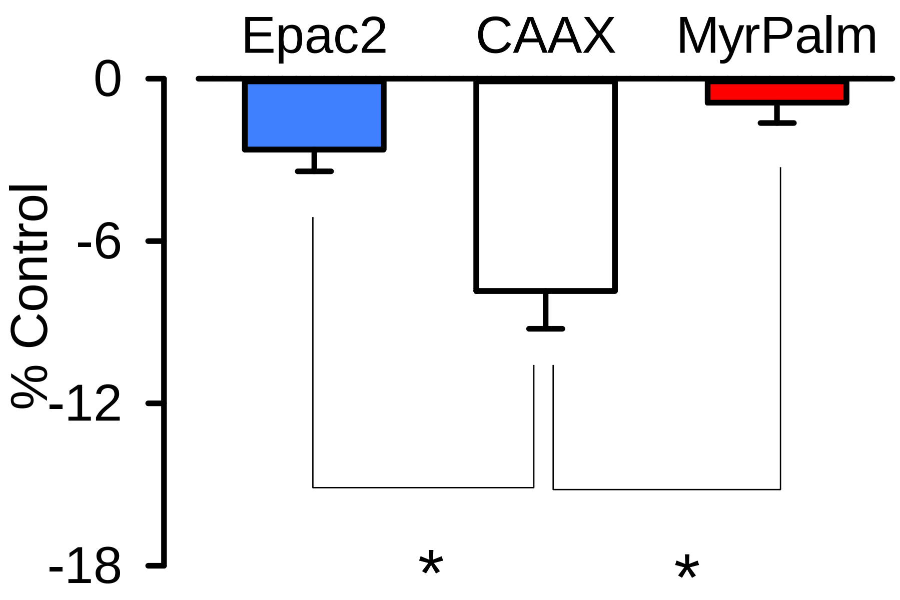


**Supplementary Figure 4. Submaximal inhibition of basal adenylyl cyclase activity leads to greater reduction of cAMP activity in non-raft associated membrane domains.** Summary of average FRET ($\Delta R/R_0$) responses to submaximally inhibiting concentration of adenylyl cyclase inhibitor MDL12330A (MDL; 30 µM) cells expressing Epac2-camps (n/N = 8/3, blue bar), Epac2-CAAX (n/N = 10/6, white bar), and Epac2-MyrPalm (n/N = 10/4, red bar). Epac2-CAAX response to 30 µM MDL was significantly different from Epac2-camps and Epac2-MyrPalm responses (*$p < 0.05$, Kruskal-Wallis one-way ANOVA on Ranks followed by Dunn's test for pairwise multiple comparisons).
